# Multiple-level validation identifies *PARK2* in the development of lung cancer and chronic obstructive pulmonary disease

**DOI:** 10.18632/oncotarget.9954

**Published:** 2016-06-13

**Authors:** SeungBaek Lee, Jun She, Bo Deng, JungJin Kim, Mariza de Andrade, Jie Na, Zhifu Sun, Jason A. Wampfler, Julie M. Cunningham, Yanhong Wu, Andrew H. Limper, Marie-Christine Aubry, Chris Wendt, Peter Biterman, Ping Yang, Zhenkun Lou

**Affiliations:** ^1^ Division of Oncology Research, Mayo Clinic, Rochester, MN, USA; ^2^ Department of Pulmonary Medicine, Zhongshan Hospital, Fudan University, Shanghai, China; ^3^ Department of Health Sciences Research, Mayo Clinic, Rochester, MN, USA; ^4^ Department of Thoracic Surgery, Institute of Surgery Research, Daping Hospital, Third Military Medical University, Chongqing, China; ^5^ Genomics Shared Resource, Mayo Clinic, Rochester, MN, USA; ^6^ Division of Pulmonary and Critical Care Medicine, Mayo Clinic, Rochester, MN, USA; ^7^ Division of Anatomic Pathology, Mayo Clinic, Rochester, MN, USA; ^8^ Department of Medicine, Pulmonary Division, University of Minnesota, Minneapolis, MN, USA; ^9^ Department of Medicine, Pulmonary Section, Minneapolis VA Medical Center, Minneapolis, MN, USA

**Keywords:** lung cancer, chronic obstructive pulmonary disease, PARK2, inflammation

## Abstract

An important precursor to lung cancer development is chronic obstructive pulmonary disease (COPD), independent of exposure to tobacco smoke. Both diseases are associated with increased host susceptibility, inflammation, and genomic instability. However, validation of the candidate genes and functional confirmation to test shared genetic contribution and cellular mechanisms to the development of lung cancer in patients with COPD remains underexplored. Here, we show that loss of *PARK2* (encoding Parkin) increases the expression of proinflammation factors as well as nuclear NF-κB localization, suggesting a role of *PARK2* loss in inflammation. Additional exploration showed that *PARK2* deficiency promotes genomic instability and cell transformation. This role of *PARK2* in inflammation and chromosome instability provides a potential link among Parkin, COPD and lung cancer. A further comprehensive validation of 114 informative single nucleotide polymorphism (SNP) variants of *PARK2,* in 2,484 cases and controls with well-defined lung cancer and COPD phenotypes, found rs577876, rs6455728 and rs9346917 (*p<0.01*) to be significantly associated with lung cancer development in people with COPD. Our findings support the evidence that *PARK2* might have a tumor suppressor role in the development of COPD and lung cancer.

## INTRODUCTION

Lung cancer and chronic obstructive pulmonary disease (COPD) combined, present a major cause of morbidity and mortality globally that has persisted for several decades [1–3]. These two complex diseases are closely linked to each other as shared environmental and genetic risk factors are implicated in both [4, 5]. COPD coexists in 40-70 % of patients with lung cancer and is increasingly recognized as a predecessor of lung cancer development, independent of tobacco exposure [6] and most published genetic studies focused on either lung cancer or COPD [5, 7–9]. Chronic inflammation plays a central role and key inflammatory cells such as macrophages, neutrophils, and T lymphocytes are involved in COPD [10–14]. Macrophages from COPD patients release higher levels of pro-inflammatory cytokines (TNF-α and IL-6) compared to nonsmoking control subjects [15, 16]. Inflammation and COPD together are strongly associated with lung cancer but the molecular basis for inflammation and cancer has not been established yet.

Loss of function mutations in the gene *PARK2* encoding Parkin, an E3 ubiquitin ligase, leads to death of dopaminergic neurons and causes Autosomal Recessive Juvenile Parkinsonism (AR-JP) [17, 18], the neuroprotective role of *PARK2* is linked to mitophagy [19]. Recently, *PARK2* has been suggested as a haplo-insufficient tumor suppressor gene, based on the frequent deletion or mutation in human cancer [20, 21]. Consistent with this notion, overexpression of *PARK2* in hepatocarcinoma, glioblastoma, lung cancer, breast cancer and colon cancer cell lines repressed cell growth [20–24]. Furthermore, *PARK2*-null mice displayed increased vulnerability to inflammation-induced degeneration when exposed to chronic systemic lipopolysaccharide (LPS) [25]. Cancer cell lines that harbor a deleted *PARK2* gene show resistance to TNF-α induced cell death [16]. These observations suggest a potential function of *PARK2* as a regulator of immune response. However, how *PARK2* functions in immune response and its relationship to tumorigenesis remains unclear. In this study, we investigated a potential role of *PARK2* in inflammation, COPD and lung cancer.

## RESULTS

### Loss of *PARK2* maintains higher expression of cytokines for inflammation

**Figure 1:**
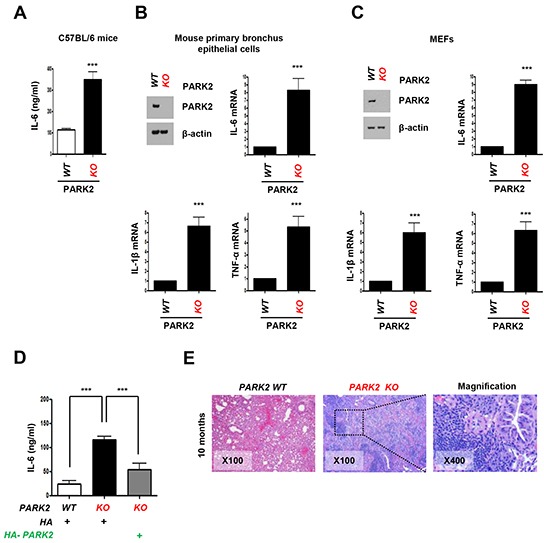
Loss of *PARK2* maintains higher expression of cytokines for inflammation **A-C.** Cytokines, IL-6, IL-1β or TNF-α protein or mRNA levels in serum from C57BL/6 mice (A), mouse primary bronchus epithelial cells (B), MEFs (C) of *PARK2* WT or KO were analyzed by ELISA or qRT-PCR. **D.** After infection with the indicated plasmids, cytokines in the cultured MEFs supernatants were determined by ELISA. Results are shown as means (±SEM), and at least three experiments were performed for all experiments. ***,*p* < 0.001 by one-way ANOVA. **E.** *PARK2 WT* and *KO* mice (*n=6*) at 10 months of age were analyzed for tumor event by immunohistochemistry. H&E staining; x100 and x400.

To assess the role of PARK2 in inflammation, we generated *PARK2* knockout (KO) in C57BL/6 mice and assessed IL-6 levels in mouse serum. Notably, IL-6 levels in *PARK2* KO mice were significantly higher than those in wild type (WT) mice (Figure 1A). Similarly, mouse primary bronchus epithelial cells and mouse embryonic fibroblasts (MEFs) from *PARK2* KO mice produced more IL-6 than controls (Figure 1B and 1C). Furthermore, the expression of other inflammation markers, IL-1β and TNF-α also increased in *PARK2* deleted cells (Figure 1B and 1C). In addition, the increases caused by *PARK2* KO were reversed by exogenous expression of WT *PARK2* (Figure 1D). These results suggest that PARK2 has anti-inflammation functions. We also performed immunohistochemistry (IHC) in *PARK2* wild type (WT) and knockout (KO) mouse at 10 months (Figure 1E). *PARK2* KO mice showed increased inflammation phenotypes, as evinced by densely packed plasma cells around lung bronchia. This data suggested that *PARK2* deficiency leads to increased inflammation.

### Overexpression of *PARK2* inhibits localization of the nuclear NF-κB for inflammation

**Figure 2:**
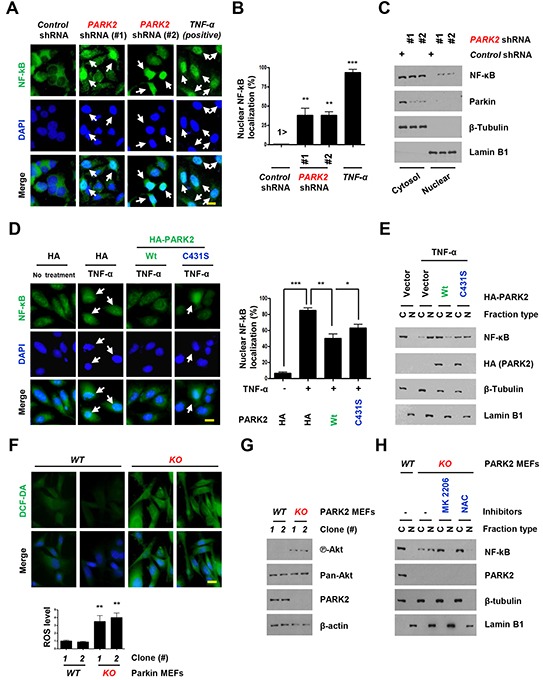
Overexpression of *PARK2* inhibits localization of the nuclear NF-κB for inflammation **A-C.** After infection with the indicated shRNAs, Primary and immortalized (BEAS-2B) human bronchial epithelial cell lysates were fixed for immunofluorescence assay or blotted with the indicated antibodies. (A) Representative images of the cells with indicated localization of the nuclear NF-κB (*white arrows*). Cells were fixed and stained with DAPI. TNF-α-treated cells were shown as positive controls. Scale bar, 20 μm. (B) Analysis of numerical nuclear NF-κB events. 100 cells were counted in each experiment. **, *p* < 0.01 and ***,*p* < 0.001 by one-way ANOVA. (C) Immunoblot analysis with indicated antibodies after nuclear fractionation. **D.** After transfection with the indicated plasmids, Beas-2B cells were selected by G418 to select stable transfectants, cells were treated with TNF-α and cells lysates were fixed for immunofluorescence assay or cell lysates were analyzed immunobloting. Representative images of the cells with indicated localization of the nuclear NF-κB (*white arrows*). Cells were fixed and stained with DAPI. TNF-α-treated cells were shown as negative controls. Scale bar, 20 μm. Quantification of the nuclear NF-κB localization. Results are shown as means (±SEM), and at least three experiments were performed for all experiments. *, *p* < 0.05, **, *p* < 0.01 and ***, *p* < 0.001 by one-way ANOVA. **E.** After nuclear fractionation, NF-κB, Lamin B1 and β–tubulin expression was measured by immunoblot. Lamin B1, nuclear marker; β–tubulin, cytosol marker. **F.** Cells were stained with DCF-DA, fixed and analyzed by immunofluorescence. **G.** Cells were harvested and analyzed by immunobloting. **H.** Cells were treated with the indicated chemicals, MK 2206 (an allosteric Akt inhibitor) or NAC (ROS scavenger). After nuclear fractionation, NF-κB, Lamin B1 and β–tubulin expression was measured by immunoblot. Lamin B1, nuclear marker; β–tubulin, cytosol marker.

Nuclear factor kappa B (NF-κB) is a broadly expressed transcription factor that induces cytokines and immunoglobulin (Ig) gene expression in COPD-related inflammation [26]. Although NF-κB is present in its inactive state in the cytoplasm, its p50–p65 heterodimer translocates to the nucleus and binds the DNA (at the promoter region) when NF-κB is activated by carcinogens, tumor promoters, inflammatory cytokines, and other chemotherapeutic agents [27]. To understand PARK2′s role in inflammation, we monitored NF-κB localization in *PARK2*-depleted primary human bronchial epithelial BEAS-2B cells using fluorescence microscopy (Figure 2A and 2B) and immunoblot (Figure 2C). The depletion of *PARK2* led to increased nuclear NF-κB localization in the absence of stimuli (Figure 2A, 2B and 2C). In addition, TNF-α treatment-induced nuclear NF-κB translocation was significantly blocked by the expression of WT *PARK2* (Figure 2D and 2E). Interestingly, expression of mutant *PARK2* that inactivates its E3 ligase has a partial effect in blocking NF-κB translocation. These results demonstrate that *PARK2* suppresses NF-kB activation in an E3 ligase-dependent and -independent manner.

We next explored how PARK2 suppresses NF-kB activation. Several papers reported involvement of PARK2 in the antioxidant defense [28, 29]. We confirmed increased Reactive oxygen species (ROS) levels in *PARK2* KO MEFs (Figure 2F). ROS has been linked to increased Akt activation, and NF-kB activation [30–33]. Consistent with this, we found increased Akt S473 phosphorylation in *PARK2* KO MEFs (Figure 2G). Treatment of *PARK2* KO MEFs with NAC, ROS scavenger or MK 2206, Akt inhibitor (Figure 2H) decreased NF-kB translocation, suggesting that PARK2 suppresses NF-kB signaling through ROS/Akt regulation [30, 32, 33].

### Deletion of *PARK2* induced chromosome stability and tumor initiation

**Figure 3:**
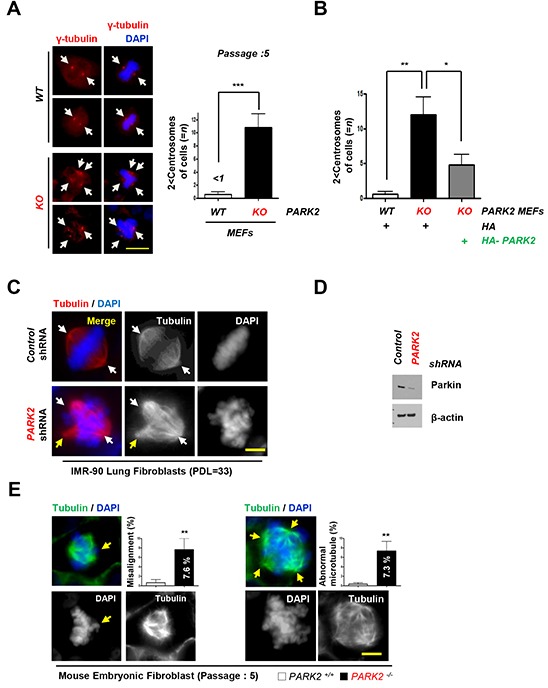
Deletion of *PARK2* induced chromosome instability **A.** *PARK2* deficiency results in centrosome amplification in MEFs at passage 5. (*left*) Immunofluorescence staining shows impaired mitoses in MEFs depleted of *PARK2*. Red, γ-tubulin; White arrows, centrosome in mitosis. The scale bar is 20 μm. (*right*) Quantification of 2 < centrosomes cells. ***, *p* < 0.001 by one-way ANOVA. *n*=10. **B.** After infection with the indicated plasmids, MEFs lysates were fixed for immunofluorescence assay. Quantification of 2 < centrosomes cells using γ-tubulin. Results are shown as means (±SEM), and at least three experiments were performed for all experiments. *, *p* < 0.05 and **, *p* < 0.01 by one-way ANOVA. **C.** Immunofluorescence staining shows impaired mitoses in IMR 90 lung fibroblast cells depleted of *PARK2*. White arrows, centrosome in mitosis. Yellow arrows, abnormal chromosome. The scale bar is 20 μm. **D.** Cell were harvested at indicated times and analyzed by immunobloting. **E.** Impaired mitoses including multipolar spindles, misalignment and abnormal microtubule in *PARK2* knockout (KO) MEF cells. Yellow arrows, abnormal chromosome. The scale bar is 20 μm. Results are shown as means (±SEM), and at least three experiments were performed for all experiments. **, *p* < 0.01 by one-way ANOVA.

In addition to increased inflammation, *PARK2* deficiency also resulted in genomic instability [15, 21, 34]. We found that loss of *PARK2* induced centrosome duplication. Using an antibody to γ-tubulin, we compared the number of centrosome in *PARK2* KO and wild-type MEFs at Passage 5. Our data showed that 11 % of *PARK2 KO* cells contained three or more centrosomes, whereas very few *WT* MEFs have abnormal centrosomes (Figure 3A). The reconstitution of *PARK2* KO cells with *PARK2* corrected the centrosome abnormities (Figure 3B). Similar results were obtained using IMR-90 lung fibroblasts (PDL=33) or MEFs supporting the idea that *PARK2* deficiency results in genomic instability (Figure 3C, 3D and 3E).

**Figure 4:**
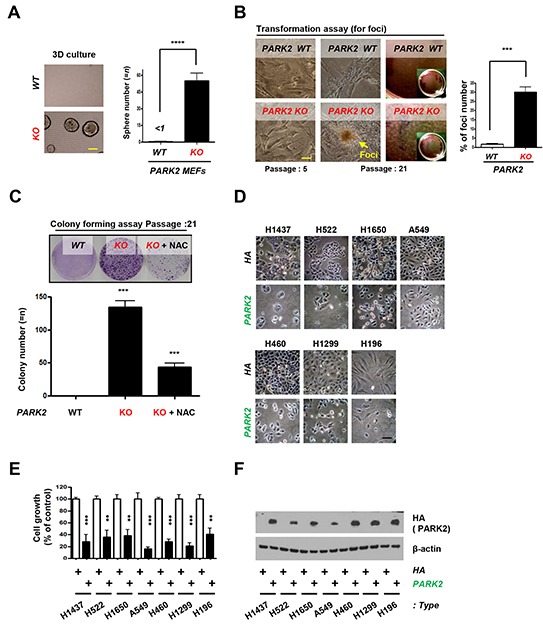
PARK2 loss leads to tumor initiation and overexpression of *PARK2* inhibits cell proliferation in lung cancer cell lines **A.** Primary MEFs were applied on 3-D cultures system for 2 weeks and counted the sphere of the cells. (*top*) Representative images indicate the sphere cells. (*bottom*) Quantification of the sphere number. ****, *p* < 0.0001 by one-way ANOVA. *n*=6. (**B.** and **C.**) *PARK2* WT and KO MEFs were analyzed for foci formation at passage 5 and 21 (B), and colony formation at passage 21 **C.** The scale bar is 20 μm. Results are shown as means (±SEM), and at least three experiments were performed for all experiments. NAC, ROS scavenger. ***, *p* < 0.001 by one-way ANOVA. **D.** The growth of lung cancer cell lines (H1437, H522, H1650, A549, H460, H1299 and H196) was inhibited through infection with *PARK2*. Cells were transfected with the indicated plasmids, and then cell lysates were blotted with the indicated antibodies. **E.** The percentage of lung cancer cell growth compared to controls after transfected *PARK2*. Results shown as means (± SEM), and at least three experiments were performed for all experiments. **, *p* < 0.01 and ***, *p* < 0.001 by one-way ANOVA. **F.** PARK2 and β–actin expression was measured by immunoblot.

Our results suggest that *PARK2* deficiency results in increased inflammation and genomic instability, both of which are potential drivers of tumorigenesis [20, 21, 23, 25]. To confirm whether *PARK2* contributes to tumorigenesis in ex-vivo models, we seeded *PARK2* WT or KO MEFs in 3-D organoid culture system. Culture of cells as three-dimensional (3-D) aggregates can enhance *in vitro* tests for basic biological research as well as mimics tissue organization condition *in vivo* [35, 36]. We found that *PARK2* KO MEFs, but not WT MEFs, formed colonies in 3-D culture (Figure 4A). Furthermore, we found that *PARK2* knockout MEFs became transformed and promoted cancer cell growth by foci and colony formation assay, where the frequency of foci number and colony number were increased (Figure 4B and 4C), suggesting a role of *PARK2* loss in tumor initiation. Treatment of *PARK2*-deficient MEFs with N-acetyl-l-cysteine (NAC), ROS scavenger, significantly decreased colony formation, suggesting that ROS is a contributing factor in cellular formation induced by *PARK2* loss. We next examined the potential role of *PARK2* on the growth of lung cancer cells. After the *PARK2* gene was expressed in seven lung cancer cell lines (H1437, H522, H1650, A549, H460, H1299 and H196), the growth of tumor cell lines was significantly inhibited compared to controls (Figure 4D, 4E and 4F), which suggests that *PARK2* overexpression plays a critical role in the inhibition of lung cancer cell proliferation [28]. Multiple *PARK2* mutations have also been identified in human lung cancer (Supplementary Table S1). These results suggest that *PARK2* deficiency might contribute to lung cancer development.

### *PARK2* as a potential target to reduce the risk of COPD and lung cancer

**Figure 5:**
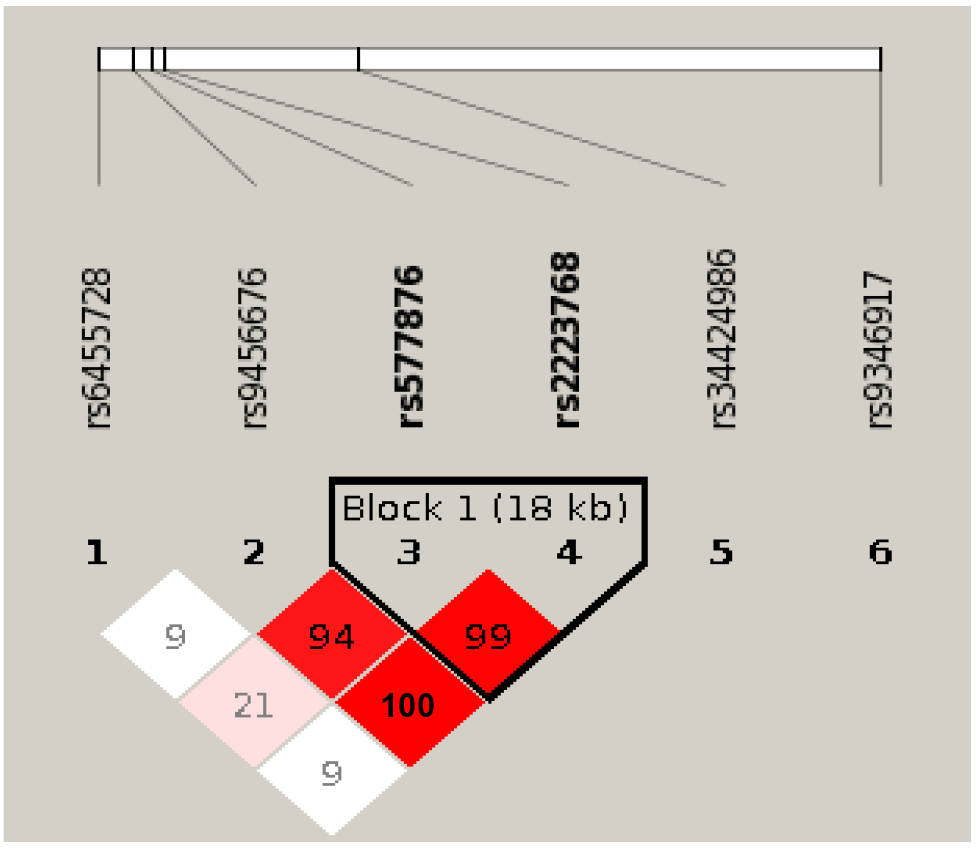
Linkage disequilibrium (LD) plot of 6 SNPs in *PARK2* gene Among the six SNPs, five (SNPs 1-4 and 6) are tested significant in our Model 4 that comparing cases with lung cancer and chronic obstructive pulmonary diseases (COPD) to controls with COPD only. One SNP rs34424986, which was recently reported from an independent study [24] but not included on GWAS panels, is shown relative position among the five tested significant SNPs. Numbers shown in blocks are D' values (i.e., D'x100; 99 means D' = 0.99), which measures the strength of LD between two neighboring SNPs. The higher of D' value, the more statistically associated between two SNPs. Because the high LD between rs9456676, rs577876 and rs2223768, final results of penalized regression model removed two SNPs (rs9456676 and 2223768). LOD, means logarithm of odds score, is a statistical test used in genetic linkage analysis. **Legends**: White - D'<1, LOD <2; Pink shades - D'<1, LOD ≥ 2; Red - D' =1, LOD ≥ 2;None: SNPs not correlated.

As *PARK2* deficiency results in both increased inflammation and genomic instability [37–39], we assessed a potential role of *PARK2* in lung cancer and COPD through a comprehensive validation study of 114 informative single nucleotide polymorphism (SNP) variants of *PARK2* (Supplementary Table S2) in 2,484 cases and controls with well-defined lung cancer (LC) and COPD phenotypes (Table 1). For specific comparisons, we assigned the four groups into the six models as follows: Model 1: LC+COPD+ vs. LC-COPD- (cases with both compared to controls with neither); Model 2: LC+COPD- vs. LC-COPD- (cases with LC compared to controls with neither); Model 3: LC-COPD+ vs. LC-COPD- (cases with COPD compared to controls with neither); Model 4: LC+COPD+ vs. LC-COPD+ (cases with both compared to controls with COPD); Model 5: LC+ vs. LC- (LC cases compared to LC-free controls, adjusting for COPD and other covariates including age, sex, and smoking history); and Model 6: COPD+ vs. COPD- (COPD cases compared to COPD-free controls, adjusting for LC and other covariates including age, sex, and smoking history). After using two methods of minimizing false positive results and a penalized logistic regression analysis [40–42], rs577876, rs6455728 and rs9346917 (*p<0.01*) were confirmed to be associated with lung cancer development in people with COPD (in Model 4 only, Table 2). Figure 5 shows the LD haplotype blocks of five SNPs that were tested significant in Model 4. Noted is rs34424986 [24, 43], which lies between rs2223468 and rs9346917.

**Table 1:** Distribution of lung cancer (LC) and chronic obstructive pulmonary diseases (COPD) in 2484 cases and controls

	COPD +	COPD -	Total
LC +	n +/+ (573)	n +/− (612)	1185
LC −	n −/+ (537)	n −/− (762)	1299
Total	1110	1374	2484

**Table 2:** Significant results of Testing 114 Informative PARK2 SNPs

*PARK2 Gene (Chromosome 6) SNP rs-ID*		Alleles(major: minor)	MAF controls^*^	MAF cases^*^	Penalized regression	
					OR	P-value
*PARK2*	rs577876	A:G	0.41	0.35	0.73	5.49×10^−4^
*PARK2*	rs6455728	G:T	0.29	0.24	0.70	3.28×10^−3^
*PARK2*	rs9346917	C:T	0.35	0.30	0.68	1.66×10^−3^

* MAF is Minor Allele Frequency calculated from the controls (LC+COPD-) and cases (LC+COPD+).

This is the first study that presents lung cancer with COPD as a separate disease entity from the lung cancer or COPD phenotype [44]. We found the *PARK2* gene associates with the phenotype of lung cancer with COPD, compared to COPD patients. Notably, altered PARK2 expression induced chromosomal instability and increased inflammation, similar to that found in the COPD phenotype. We demonstrated that *PARK2* may be a bona fide tumor suppressor gene that may be responsible for the development of progression lung cancer with COPD, suggesting *PARK2* as a potential target to reduce the risk of COPD and lung cancer. Our results evinced the biological plausibility as well as the common and unique mechanisms in lung cancer with COPD development [45].

## DISCUSSION

Our analysis of *PARK2*, located at 6q25.2-q27, revealed four significant yet independent SNPs to be associated with lung cancer with COPD. Although the function of *PARK2* in lung cancer remains unknown, we suggest that PARK2-deficiency induced inflammation and genomic instability are possible factors contributing to lung cancer. *PARK2* mutations are known to cause Parkinson's disease and have also been identified in human malignancies, including lung cancer, and are associated with the potential to inhibit cancer cell growth *in vitro* [21], suggesting that *PARK2* may function as a tumor suppressor gene. Additionally, a SNP in *PARK2* was associated with a decline in forced expiratory volume in 1 second (FEV1) and a recurrent *PARK2* mutation was recently reported [24, 46]. Our study demonstrated that *PARK2* expression significantly inhibited cell growth in lung cancer cells lines compared to controls, while abnormal inflammation and cell division was induced by *PARK2* deficiency. This result could be interpreted as a role in cancer initiation, progression or both. As inflammation and chromosomal instability are a driving forces for tumor initiation, it is possible that *PARK2* mutation/loss may also involved in lung cancer progression and prognosis (Supplementary Figure S1).

In conclusion, our multi-level validation study suggests a critical role of *PARK2* in the development of lung cancer with COPD. We showed that *PARK2* inhibiting role in inflammation, lung cancer cell growth, and the chromosomal instability that might contribute to tumor initiation when *PARK2* expression is altered. We used strict inclusion criteria in the study. Lung cancer diagnoses were re-confirmed by pulmonary pathologists and COPD was confirmed by lung function testing. The definition of COPD phenotype based on lung function tests minimizes the phenotypic heterogeneity caused by the use of clinical records or patient reported symptoms. As a result, the reliability and validity of our study is sound. These findings have advanced the current understanding of the development of lung cancer with COPD, and the progression from COPD to lung cancer, potentiating the discovery of new targets for prevention, early diagnosis, and treatment.

## MATERIALS AND METHODS

### Mouse strains and MEFs

All animal procedures were approved by the Institutional Animal Care and Use Committee which also approved experimental procedures. Mouse strains were described previously [34, 47]. *PARK2* KO C57BL/6 (6–8 weeks old, female) mice were purchased from the Jackson Laboratory (Bar Harbor, ME, USA) and mated. Mouse embryonic fibroblasts were isolated from individual day 11.5-13.5 (E11.5 - E13.5) embryos by uterine dissection. Each embryo was washed gently with 1x PBS (pH 7.2), followed by removal of the mouse embryo's head and liver. The embryo body was suspended in 0.5 ml of 0.25 % Trypsin-EDTA, and then forced through a 1 ml syringe with an 18-gauge needle. The tissue homogenate was incubated for 30 min at 37°C, triturated by drawing the suspension through a pipette, and then evenly divided into two 10 cm tissue culture dishes in Dulbecco's modified Eagle's medium (DMEM) with 15 % fetal bovine serum (FBS). Early-passage MEFs (passage 1–5) were used for all experiments, and at least three lines were examined for all studies. Animals were housed in a pathogen-free barrier environment throughout the study.

### Cells and cell lines and reagent

All cell lines were sourced from the American Type Culture Collection (ATCC, Manassas, VA). These included seven lung cancer cells, H1437, H522, H1650, A549, H460, H1299 and 196, and one normal human lung fibroblast cell line, IMR-90. c. For the human lung fibroblast IMR-90 cells ranging from 29 to 34 in population doubling level (PDL) were used. Cell lines were maintained in Eagle's minimal essential media (EMEM, Gibco-Invitrogen, Grand Island, NY) and contained 10 % (15 % for IMR-90 cells) heat-inactivated FBS (Gibco-Invitrogen), sodium bicarbonate (2 mg/ml; Sigma-Aldrich, St Louis, MO), penicillin (100 units/ml), and streptomycin (100 μg/ml; Gibco-Invitrogen). An ELISA kit (R&D Systems) was used to assay IL-6 production, according to the manufacturer's instructions. The concentrations of IL-6 were normalized to the cell numbers in the cell culture. 2′,7′- dichlorofluorescein diacetate (DCF-DA) was obtained from Molecular Probes. MK 2206 was obtained from selleckchem. N-acetylcystein (NAC) was obtained from Gibco-Invitrogen.

### Immunoblotting and antibodies

For immunoblotting, extraction of proteins from cultured cells with a modified buffer was followed by immunoblotting with corresponding antibodies. Briefly, protein samples were fractionated on 10 - 15 % SDS polyacrylamide gels and electroblotted onto Hybond ECL nitrocellulose membranes (Amersham Pharmacia Biotech) using a semidry transfer apparatus (Bio-Rad). Rabbit polyclonal antibodies recognizing PARK2 (ab15954), were obtained from Abcam. Mouse monoclonal antibody recognizing PARK2 (sc-32282) and Lamin B1 (sc-20682) were purchased from Santa Cruz Biotechnology. Rabbit polyclonal antibody recognizing NF-κB (sc-372) was obtained from Santa Cruz Biotechnology. Anti-β-tubulin and HA mouse antibodies were purchased from Sigma.

### Immunofluorescence

For immunofluorescence staining, MEF or IMR90, Beas-2B cells were plated on glass coverslips and transfected with the indicated constructs. Cells were then fixed in 3.7 % Paraformaldehyde for 10 min at room temperature and stained using standard protocols. Immunofluorescence images were taken using fluorescent microscopy (Nikon Microscope, Melville, New York).

### Plasmid and gene silencing by lentiviral shRNAs

The pMX retroviral vector containing the human cDNAs for HA-PARK2 was obtained from Addgene (plasmid 38248, Cambridge, MA). *PARK2* vectors were obtained from Sigma-Aldrich and Open Biosystems.

**Table:** 

Company	Species	Clone Set ID	Names	Target sequence (5′- -3′)
Open Bio.	Human	NM_013988	84517	5′-GAGAGAGTTCTCACATTTAAT-3′
Open Bio.	Human	NM_013988	84518	5′-ACTCACTAGAATATTCCTTAT-3′
Open Bio.	Human	NM_013988	84520	5′-GAACGTTTAGAAATGATTTCAAA-3′
Sigma (TRC1)	Human	NM_013988	2399	5′-CGTGAACATAACTGAGGGCAT-3′
Sigma (TRC1)	Human	NM_013988	341	5′-CGCAACAAATAGTCGGAACAT-3′
Sigma (TRC1)	Human	NM_013988	425	5′-CGTGATTTGCTTAGACTGTTT-3′
Sigma (TRC1)	Human	NM_013988	434	5′-CTTAGACTGTTTCCACTTATA-3′
Sigma (TRC1)	Human	NM_013988	872	5′-CTCCAAAGAAACCATCAAGAA-3′

### Colony formation or foci assay

For colony formation or foci assay, early-passage MEFs (passage 5) cells were plated at low density into 60-mm cell culture plates. When sufficient colonies were visible, typically after 2-3 weeks, cells were washed twice in PBS before fixing in ice-cold 70 % methanol for 30 min, stained by 0.2 % Crystal violet for 2-3 h. The following day cells were rinsed in PBS and air-dried.

### Cell fractionation assay

Cell preparations were described previously. [31] The Nuclear extract kit (California, USA) was used to perform cellular fractionation in accordance with the manufacturer's instructions. The purity of the extract was confirmed by western blot analysis against anti-cytosol-specific-tubulin-actin (Sigma-Aldrich) or anti-nuclear-specific-Lamin B1.

### 3-dimensional (3-D) organoid culture Assay

For 3-dimensional (3-D) assays, *PARK2* WT or KO MEFs were cultured on NanoCulture plates (SCIVAX Corp.). After seeding cells, cell were cultured in same condition with 2-D culture and observe cell images and recover sample 0, 3, 5, 7 days after seeding. Cells were lysed in Spheroid Lysis Buffer (Scivax).

### Phenotype definitions

Lung cancer cases were pathologically diagnosed as defined by the World Health Organization [48]. COPD diagnosis was based on the criteria of the Global Initiative for Chronic Obstructive Lung Disease (GOLD) [49], i.e., subjects with a FEV1/FVC < 70 % by lung function test (PFT) as COPD+ regardless of sub-groups (emphysema and/or chronic bronchitis). All subjects provided written informed consent, and the study protocol was approved by the Mayo Clinic Institutional Review Board.

### Experimental design for PARK2 SNPs validation

#### Subject selection and detailed phenotyping

A total of 2,484 well-defined subjects were included in the current study following a 4-step sampling strategy in order to minimize the confounding factors and maximize the uncommon groups: [1] enriching never-smoking cases; [2] balancing three phenotype groups, i.e., COPD only (COPD+), lung cancer only (LC+), both COPD and lung cancer (LC+COPD+) and controls (LC-COPD-); [3] matching on current vs. former smoking history with gender and age; and [4] for former smokers, matching on quitting years. To minimize the heterogeneity due to population stratification, we restricted our study to subjects of European descent [9, 44, 50–52]. In total, the study consisted of 612 with LC+, 537 COPD+, 573 LC+COPD+, and 762 controls; yielding 1,110 COPD cases and 1,185 lung cancer cases.

### Genome wide analysis and candidate SNPs validation

Using GWAS Data from two Illumina platforms (OmniExpress, 610k and 370k Human BeadChips; Illumina, San Diego, California, USA) we performed genome-wide association analyzes using the models described above Concordance between platforms was > 99.5 %. We removed samples with genotyping call rates < 95 % from structure analysis. SNP quality control removed SNPs with a call rate < 95 %, minor allele frequencies (MAF) < 0.01, Hardy-Weinberg equilibrium test *p* < 0.0001. A quantile-quantile plot was generated using logistic regression for additive genetic model, and 4 sets of above-defined group comparisons of genomic inflation factors (λ_GC_) were 1.006, 1.011, 0.98122, 0.98185, respectively. A total of 114 candidate SNPs for the *PARK2* gene were matched in our genotyping platforms, after removing the redundant SNPs using linkage disequilibrium (LD) test (r^2^≥0.8) [53].

### Real time PCR

RNA preparation, cDNA, and qRT-PCR were described previously [31]. The following primers were used:

Primers Used for Real-Time PCR

IL-6 Primer sequence: Forward 5′-CCTGCGTTTAAATAACATCAGCTTTAGCTT-3′,

Reverse 5′-GCACAATGTGACGTCGTTTAGCATCGAA-3′,

IL-1β Primer sequence: Forward 5′-CACAGCAGCA CATCAACAAG-3′,

Reverse 5′-GTGCTCATGTCCT CATCCTG-3′,

TNF-α Primer sequence: Forward 5′-CCAGCCAGCAGAAGCTCCCTCAGCGAG-3′,

Reverse 5′-GCGGATCATGCTTTCTGTGCTCATGGTGTC-3′

### Statistical analysis

Each assay was performed in triplicate and independently repeated at least three times. The results are presented as mean ± standard error of mean (SEM). Statistical analyses were performed using GraphPad Prism software (version 4.02; GraphPad Software, San Diego, CA). One-way analysis of variance (ANOVA) followed by T-test was used to compare the results. A difference was considered significant if *P*<0.05. Statistical significance was defined as *p*<0.05 (^*^), *p*<0.01(^**^), and *p*<0.001(^***^).

## SUPPLEMENTARY TABLES AND FIGURES






