# Supplementary material for: Multiple-level validation identifies PARK2 in the development of lung cancer and chronic obstructive pulmonary disease
Source: Oncotarget. 2016 Jun 13;7(28):44211–23. doi: 10.18632/oncotarget.9954 (PMC5190090; doi:10.18632/oncotarget.9954)
Supplement: Supplementary file 1 [file oncotarget-07-44211-s001.pdf]

Multiple-level validation identifies *PARK2* in the development of lung cancer and chronic obstructive pulmonary disease

SUPPLEMENTARY TABLES AND FIGURES

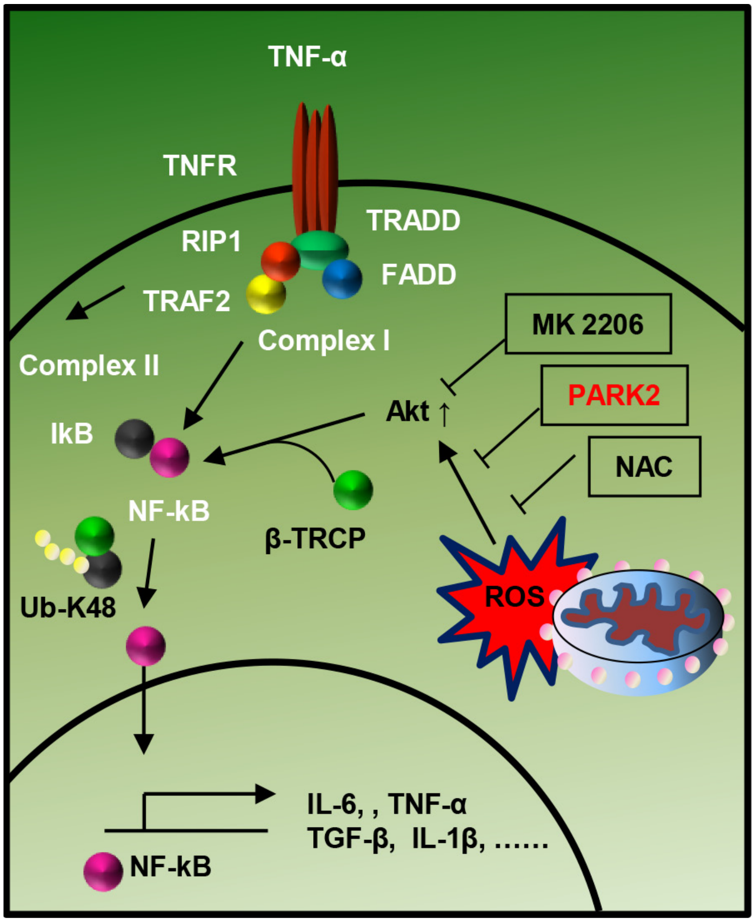

Supplementary Figure S1: Schematic model.

Supplementary Table S1: PARK2 mutants in human lung cancer. 46 Parkin mutations in cBioPortal (http://www.cbioportal.org/).

See Supplementary File 1

Supplementary Table S2: The 114 candidate SNPs selected for *PARK2* gene.

See Supplementary File 2
