# Supplementary material for: Multiple-level validation identifies PARK2 in the development of lung cancer and chronic obstructive pulmonary disease
Source: Oncotarget. 2016 Jun 13;7(28):44211–23. doi: 10.18632/oncotarget.9954 (PMC5190090; doi:10.18632/oncotarget.9954)
Supplement: Supplementary file 2 [file oncotarget-07-44211-s002.pdf]

Supplementary Table 1

Supplementary Table 1. PARK2 mutants in human lung cancer. 46 Parkin mutations in cBioPortal (http://www.cbioportal.org/).

	Cancer Study	AA change	Type	Copy #	COSMIC	Mutation Assessor	Allele Freq (T)	# Mut in Sample
LUAD-S01467	Lung adeno (Broad)	*R156**3D	Nonsense	Diploid	1		NA	279
LUAD-B02594	Lung adeno (Broad)	*X179_splice*3D	Splice	Diploid			NA	634
LUAD-GU4I3	Lung adeno (Broad)	*G336F*3D	Missense	Diploid			NA	39
LUAD-CHTN-MAD06-...	Lung adeno (Broad)	*H373D*3D	Missense	Diploid	1	Medium	NA	92
LUAD-B00915	Lung adeno (Broad)	*C449F*3D	Missense	Diploid	1	High	NA	440
WA7899	LUAD (MSKCC)	*V43L*3D	Missense	NA		Neutral	0.08	122
TCGA-78-7159-01	Lung adeno (TCGA pub)	*A138P*	Missense	ShallowDel	1	Low	0.18	258
TCGA-05-4410-01	Lung adeno (TCGA pub)	*G213V*3D	Missense	Diploid	1	Low	0.22	982
TCGA-44-7670-01	Lung adeno (TCGA pub)	*R275P*3D	Missense	ShallowDel	3	Medium	0.35	941
TCGA-78-7155-01	Lung adeno (TCGA pub)	*E322**3D	Nonsense	Diploid	1		0.27	1340
TCGA-64-5775-01	Lung adeno (TCGA pub)	*P343Q*3D	Missense	ShallowDel	3	Medium	0.74	402
TCGA-55-7907-01	Lung adeno (TCGA pub)	*E353**3D	Nonsense	Diploid	1		0.3	963
TCGA-05-4410-01	Lung adeno (TCGA pub)	*G354R*3D	Missense	Diploid	1	Neutral	0.22	982
TCGA-49-4501-01	Lung adeno (TCGA pub)	*X429_splice*3D	Splice	Diploid			0.1	72
TCGA-05-4390-01	Lung adeno (TCGA pub)	*W453L*3D	Missense	ShallowDel	1	Low	0.16	495
TCGA-49-4501-01	Lung adeno (TCGA)	*MUTATED*	Splice	Diploid			0.1	72
TCGA-78-7159-01	Lung adeno (TCGA)	*A138P*	Missense	ShallowDel	1	Low	0.18	258
TCGA-05-4410-01	Lung adeno (TCGA)	*G213V*3D	Missense	Diploid	1	Low	0.22	982
TCGA-44-7670-01	Lung adeno (TCGA)	*R275P*3D	Missense	ShallowDel	3	Medium	0.35	941
TCGA-78-7155-01	Lung adeno (TCGA)	*E322**3D	Nonsense	Diploid	1		0.27	1340
TCGA-64-5775-01	Lung adeno (TCGA)	*P343Q*3D	Missense	ShallowDel	3	Medium	0.74	402
TCGA-55-7907-01	Lung adeno (TCGA)	*E353**3D	Nonsense	Diploid	1		0.3	963
TCGA-05-4410-01	Lung adeno (TCGA)	*G354R*3D	Missense	Diploid	1	Neutral	0.22	982
TCGA-05-4390-01	Lung adeno (TCGA)	*W453L*3D	Missense	ShallowDel	1	Low	0.16	495
TCGA-66-2777-01	Lung squ (TCGA pub)	*MUTATED*	Splice	Diploid			0.16	165
TCGA-18-3416-01	Lung squ (TCGA pub)	*R97L*	Missense	Gain		Low	0.11	874
TCGA-18-3409-01	Lung squ (TCGA pub)	*P113S*	Missense	Diploid	1	Medium	0.27	2434
TCGA-34-2608-01	Lung squ (TCGA pub)	*V186I*3D	Missense	Diploid	1	Medium	0.07	116
TCGA-21-1081-01	Lung squ (TCGA pub)	*H200P*3D	Missense	Diploid	1	Neutral	0.28	171
TCGA-21-5787-01	Lung squ (TCGA pub)	*K211N*3D	Missense	Diploid	1	Medium	0.21	365
TCGA-60-2713-01	Lung squ (TCGA pub)	*I229F*3D	Missense	Diploid	2	Medium	0.04	202
TCGA-66-2754-01	Lung squ (TCGA pub)	*E300G*3D	Missense	Diploid	1	Medium	0.36	396
TCGA-66-2757-01	Lung squ (TCGA pub)	*Q347H*3D	Missense	Gain	2	Low	0.52	209
TCGA-66-2763-01	Lung squ (TCGA pub)	*A405P*3D	Missense	ShallowDel	1	Low	0.43	332
TCGA-18-3409-01	Lung squ (TCGA)	*P113S*	Missense	Diploid	1	Medium	0.27	2505
TCGA-66-2777-01	Lung squ (TCGA)	*X138_splice*	Splice	Diploid			0.16	166
TCGA-34-2608-01	Lung squ (TCGA)	*V186I*3D	Missense	ShallowDel	1	Medium	0.07	132
TCGA-21-1081-01	Lung squ (TCGA)	*H200P*3D	Missense	Diploid	1	Neutral	0.28	171
TCGA-21-5787-01	Lung squ (TCGA)	*K211N*3D	Missense	DeepDel	1	Medium	0.21	359
TCGA-60-2713-01	Lung squ (TCGA)	*I229F*3D	Missense	Diploid	2	Medium	0.04	220
TCGA-66-2754-01	Lung squ (TCGA)	*E300G*3D	Missense	ShallowDel	1	Medium	0.36	270
TCGA-22-4593-01	Lung squ (TCGA)	*D346N*3D	Missense	Diploid	1	Low	0.04	166
TCGA-66-2757-01	Lung squ (TCGA)	*Q347H*3D	Missense	Gain	2	Low	0.52	229
TCGA-66-2763-01	Lung squ (TCGA)	*A405P*3D	Missense	ShallowDel	1	Low	0.43	359
134430	Lung SC (JHU)	*C95S*	Missense	NA	1		NA	150
2334201	Lung SC (JHU)	*T222A*3D	Missense	NA	2		NA	173
